# An Evaluation of the Diagnostic Accuracy of [68Ga]Ga-PSMA-11 vs. [18F]F-PSMA-1007 PET/CT for Lymph Node Staging in Patient Candidates for Radical Prostatectomy and Lymph Node Dissection: A Single Institutional Analysis

**DOI:** 10.3390/diagnostics15121492

**Published:** 2025-06-12

**Authors:** Paola Arena, Vittorio Fasulo, Fabrizia Gelardi, Nicola Frego, Jelena Jandric, Davide Maffei, Pier Paolo Avolio, Marco Paciotti, Giuseppe Chiarelli, Fabio De Carne, Filippo Dagnino, Andrea Piccolini, Egesta Lopci, Rodolfo Hurle, Alberto Saita, Arturo Chiti, Massimo Lazzeri, Laura Evangelista, Nicolò Maria Buffi, Paolo Casale, Giovanni Lughezzani

**Affiliations:** 1 Department of Biomedical Sciences, Humanitas University, Via Rita Levi Montalcini 4, Pieve Emanuele, 20027 Milan, Italy; paola.arena06@gmail.com (P.A.); vittorio.fasulo@humanitas.it (V.F.); giovanni.lughezzani@hunimed.eu (G.L.); 2 IRCCS Humanitas Research Hospital, Via Manzoni 56, 20089 Rozzano, Italy

**Keywords:** PSMA, prostate cancer, nodal staging, 68Ga, 18F, prostatectomy

## Abstract

**Background/Objectives**: This study evaluates and compares the diagnostic accuracy of [68Ga]Ga-PSMA-11 and [18F]F-PSMA-1007 for lymph node staging in patients with prostate cancer (PCa) scheduled for robot-assisted radical prostatectomy (RARP) and lymphadenectomy (LND). **Methods**: We retrospectively reviewed prospectively collected data on patients referred to our hospital from October 2020 to January 2023. We included all patients who underwent [68Ga]Ga-PSMA-11 or [18F]F-PSMA-1007 PET/CT for primary staging and subsequently had RARP with concomitant LND. The maximum standard uptake value (SUVmax) for lymph nodes (LNs) and the SUV node-to-background ratio were reported. Two different cut-off values for the SUV node-to-background ratio (i.e., ≥2 vs. <2 and ≥15.5 vs. <15.5) were used to evaluate the diagnostic performance of both tracers. The first cut-off was empirically chosen, while the second was based on Liu’s method. **Results**: A total of 156 patients were included (median age: 67 years). Among them, 83 underwent [68Ga]Ga-PSMA-11 and 73 underwent [18F]F-PSMA-1007 PET/CT. Suspicious lymph nodes were identified in 21 patients (13.5%). Pathological nodal involvement (pN1) was confirmed in 25 cases (16%). Of the 21 patients with suspicious pathological lymph nodes on PSMA PET/CT, 9 (42.9%) had positive nodes on the final pathology report. With an SUV node-to-background ratio cut-off of ≥2, [68Ga]Ga-PSMA-11 showed 37.5% sensitivity (SE) and 98.5% specificity(SP), while [18F]F-PSMA-1007 demonstrated 33.3% SE and 100% SP. Using the ≥15.5 cut-off, SE and SP were 31.3% and 100% for [68Ga]Ga-PSMA-11 and 11.1% and 100% for [18F]F-PSMA-1007, respectively. **Conclusions**: [18F]F-PSMA-1007 PET/CT showed, even if not statistically significantly, slightly lower SE and higher SP for nodal staging compared to [68Ga]Ga-PSMA-11 PET/CT, irrespective of the SUV ratio used.

## 1. Introduction

Prostate cancer (PCa) is the most frequently diagnosed cancer among men in over half of the countries worldwide. In 2020, an estimated 1.4 million new cases were diagnosed. PCa is also the leading cause of cancer death among men in a quarter of the world’s countries, with an estimated 375,000 deaths in 2020. Global differences in PCa incidence and mortality can be attributed to variations in screening, imaging, access to care, and healthcare infrastructure [1].

A definitive diagnosis of PCa relies on histopathological verification, but a combination of imaging techniques, including transrectal ultrasounds and magnetic resonance imaging (MRI), are currently used to optimize both PCa diagnosis and staging [2]. Consequently, the therapeutic management of PCa patients is subject to continuous innovations. Positron emission tomography/computed tomography (PET/CT) with many radiopharmaceuticals has been largely tested for PCa imaging. Currently, the main innovation in PCa imaging is represented by prostate-specific membrane antigen (PSMA).

Prostate-specific membrane antigen PET/CT, radiolabeled with 68Ga or 18F radiopharmaceuticals, is an attractive target because of its specificity for prostate tissue, even if the expression in other non-prostatic malignancies or benign conditions may cause incidental false-positive findings.

According to European guidelines, PSMA PET/CT is more sensitive in N-staging as compared to MRI, abdominal contrast-enhanced CT, or choline PET/CT. However, small LN metastases, under the spatial resolution of PET, may still be missed [3].

PSMA is a membrane glycoprotein highly upregulated in PCa cells in primary lesions, lymph nodes (LNs), and distant metastases. In contrast, PSMA is weakly expressed in normal prostate tissue [4]. The increased level of PSMA in PCa cells correlates with GS, disease stages, and androgen-independent growth, suggesting a role in prostate carcinogenesis [5,6]. Thus, PSMA is a promising target for PCa imaging and therapy [7]. PSMA radioligand PET/CT has shown higher sensitivity (SE) and specificity (SP) than conventional imaging and other radiopharmaceuticals, including choline-based agents, in both primary staging and biochemical recurrence of intermediate-to-high-risk PCa [8,9,10,11,12].

Several radiolabeled PSMA-based tracers have demonstrated efficacy for cancer detection in various clinical settings. Ga-68-labelled PSMA radioligands were the first to be developed and validated in clinical trials in humans. However, the limitations of 68Ge/68Ga generators and the high demand for PSMA PET/CT imaging have increased interest in fluorinated PSMA radiopharmaceuticals [13]. Currently, the production cost of Ga-68 is lower than that of F-18, but Ga-68 allows for fewer patients per session. Conversely, despite the higher production cost, F-18 enables the imaging of a larger number of patients per synthesis.

Considering both the cost-effectiveness and the pivotal importance of LND for staging and prognosis of these two tracers [14], we aimed to investigate the diagnostic accuracy of [68Ga]Ga-PSMA-11 and [18F]F-PSMA-1007 PET/CT for nodal staging in patients with a primary diagnosis of PCa undergoing robot-assisted radical prostatectomy (RARP) with lymph node dissection (LND). Additionally, we aimed to compare the performance of both radiopharmaceuticals in nodal staging.

## 2. Materials and Methods

### 2.1. Study Design and Selection Criteria

We retrospectively collected prospective data from patients who underwent PSMA PET/CT for primary PCa staging at our center before RARP and LND between October 2020 and January 2023. Initially, PSMA PET/CT scans were performed using Ga-68 until August 2021, after which F-18 was gradually introduced from September 2021 onwards [15]. The increasing use of F-18 is due to its “in-house” production at our institute, which allows for a higher number of daily examinations while reducing associated costs.

We included patients over 18 years old, diagnosed with PCa, and scheduled for RARP with LND at our high-volume hospital [16]. All patients provided informed consent for the acquisition of their data for research purposes upon hospitalization. PCa patients were stratified, according to the European Association of Urology (EAU) risk groups, into low-, intermediate-, and high-risk categories [14]. The preoperative risk of lymph node invasion (LNI) was assessed using the Briganti 2012 and Briganti 2019 nomograms [15,16,17,18]. Only patients with an estimated LNI risk of ≥5% or ≥7% according to these nomograms were included in the study. Patients staged or treated elsewhere, those who underwent RARP without LND, and those who refused to sign the informed consent were excluded from the analysis.

Baseline characteristics; radiological, pathological, and surgical data; postoperative outcomes; and follow-up information were collected. This study was conducted in accordance with the principles of the Declaration of Helsinki (1964). The study was approved by our local ethical committee.

### 2.2. PSMA Radioligand PET/CT Acquisition

**Figure 1:**
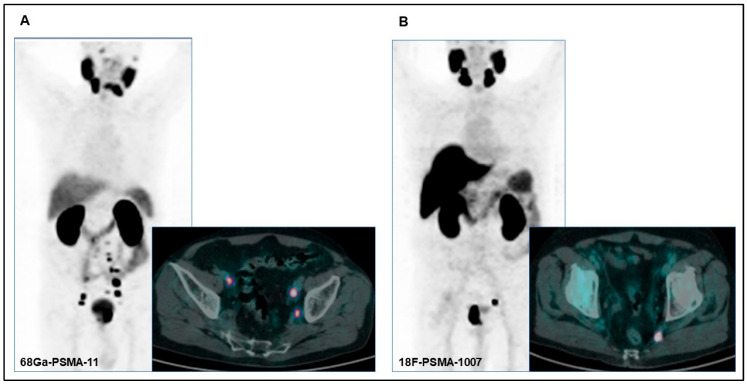
(**A**) PET/CT images of a 75-year-old patient with pathological uptake in the primary prostatic tumor and in multiple bilateral lomboaortic lymph nodes with [68Ga]Ga-PSMA-11 performed for preoperative staging (initial PSA: 7.75 ng/dL; Gleason score: 5 + 4). (**B**) PET/CT images of a 71-year-old patient with pathological uptake in the primary prostatic tumor and in a left internal iliac lymph node with [18F]F-PSMA-1007 performed for preoperative staging (initial PSA: 22 ng/dL; Gleason score: 4 + 5).

All patients included in the analysis underwent PSMA radioligand PET/CT scans with either [68Ga]Ga-PSMA-11 or [18F]F-PSMA-1007 (Figure 1A,B). PSMA radioligand PET/CT was performed according to versions 1.0 and 2.0 of the European Association of Nuclear Medicine (EANM) guidelines for prostate cancer imaging [19,20]. PET/CT images were acquired between 60 and 90 min after the PSMA radiopharmaceutical injection. All patients underwent oral hydration and bladder voiding prior to PET/CT imaging. In selected cases where high bladder activity limited the assessment of pelvic structures, furosemide (20 mg intravenously) was administered 30 min after the PSMA radioligand injection. Furosemide was administrated when clinically appropriate, avoiding patients with conditions such as benign prostatic hyperplasia or urinary incontinence. No adverse urinary effects were observed in the treated cohort.

PET/CT images were acquired using either a Siemens Biograph 6 LSO (Siemens, Erlangen, Germany) or a General Electric Discovery 690 (General Electric Healthcare, Waukesha, WI, USA) PET/CT scanner. All PET images were corrected for attenuation using the acquired CT data.

### 2.3. PSMA Radioligand PET/CT Imaging Interpretation and Analysis

PET/CT images were qualitatively and semiquantitatively analyzed by two experienced nuclear medicine physicians using a Xeleris™ workstation (General Electric Healthcare, Waukesha, WI, USA). For a qualitative analysis, PET/CT images were defined as negative if no area of increased radiopharmaceutical uptake was observed compared to the background. The criterion for positivity was at least one abnormal area of radiopharmaceutical uptake outside the physiological distribution or that higher than the surrounding physiological activity.

A quantitative analysis of the pathological lymph nodes (LNs) and background was performed manually using a spherical volume of interest of 1.8 cm^3^. The left gluteal muscle served as the reference for background uptake. Maximum standard uptake values (SUVmax) of pathological LNs were recorded for all patients. In cases with multiple pathological LNs, the node with the highest uptake was considered. The uptake of the PSMA radioligand in the pathological findings was corrected using the SUVmax of the background, and the corresponding ratios were calculated. Two different cut-offs (≥2 vs. <2 and ≥15.5 vs. <15.5) for the lymph node-to-background ratio were used to evaluate the performance of both radiopharmaceuticals. The first cut-off value was determined empirically, and the second was based on Liu’s method for optimal cut-point selection [21].

### 2.4. Surgical Technique and Pathological Analysis

All surgical teams involved in this study have extensive expertise in robot-assisted surgery, and all procedures were performed by two expert robotic surgeons. Each surgery was conducted using the Da Vinci Xi surgical system (Intuitive Surgical^®^, Sunnyvale, CA, USA). A transperitoneal approach was consistently adopted, and the LND template included the removal of nodes overlying the external iliac vessels and the internal iliac artery, as well as nodes located within the obturator fossa. Right and left pelvic nodes were separately sent for histological examination. The preoperative risk of LNI was calculated using the Briganti 2012 nomogram for all patients. For those who had undergone multiparametric MRI (mpMRI) and targeted biopsy or a combination of targeted and random biopsy, both the Briganti 2012 and 2019 nomograms were utilized [17,18].

All samples were examined by two in-house expert uro-pathologists. The postoperative Gleason score (GS), histotype, pathological staging, number and location of positive nodes, and tumor volume were reported in the definitive pathological report.

### 2.5. Statistical Analysis

Data were represented using descriptive statistical analysis. Quantitative variables included measures of central tendency and dispersion (median and interquartile range [IQR]), while qualitative variables were described as frequencies and percentages. Categorical variables were compared using the Chi-square test, and continuous variables were compared using the Wilcoxon rank sum test.

SE, SP, negative predictive values (NPVs), positive predictive values (PPVs), accuracy, and their 95% confidence intervals (CIs) were calculated to assess the overall performance of PET/CT with PSMA for preoperative primary staging in terms of lymph node involvement status. These metrics were calculated separately for each PSMA radioligand ([68Ga]Ga-PSMA-11 and [18F]F-PSMA-1007). Performance was evaluated as a dichotomous variable (negative vs. positive) and by using two different SUV-to-background ratio cut-offs (≥2 vs. <2 and ≥15.5 vs. <15.5). All analyses were conducted on a per-patient basis. Given the limited number of patients with true positive lymph node findings on PSMA PET/CT, no formal statistical comparisons were conducted between the diagnostic performance metrics of the two tracers. Instead, descriptive statistics and 95% confidence intervals were reported to reflect the uncertainty associated with estimates of sensitivity, specificity, PPVs, and NPVs.

Additionally, the performance of each agent was assessed, compared, and stratified by PCa risk group. All tests were conducted at a significance level of *p* < 0.05. All statistical analyses were performed using STATA^®^ 16.1 (StataCorp, College Station, TX, USA).

## 3. Results

### 3.1. Baseline Characteristics

Overall, we enrolled 156 patients. The median age was 67 years (IQR 60–72). Among these, 28 (17.9%) patients had a family history of PCa, and the median initial PSA was 7.60 ng/mL (IQR 6.00–14.3). According to the EAU risk group classification, 2 (1.28%) patients were categorized as low-risk, 78 (50%) as intermediate-risk, and 76 (48.7%) as high-risk PCa. All patients were treated with RARP and LND based on guidelines and Briganti’s nomogram. Of the total cohort, 83 (53.2%) patients underwent [68Ga]Ga-PSMA-11 PET/CT, and 73 (46.8%) underwent [18F]F-PSMA-1007 PET/CT. Baseline characteristics for the overall group and subgroups are reported in Table 1.

### 3.2. Nodal Staging Assessment

Positive lymph nodes were observed on PSMA PET/CT in 21 (13.5%) patients: 9 (42.9%) of these underwent [68Ga]Ga-PSMA-11 PET/CT, and 12 (57.1%) underwent [18F]F-PSMA-1007 PET/CT. The median number of surgically removed lymph nodes was 14 (IQR 11–19), with 8 (IQR 5–10) from the right side and 7 (IQR 5–9) from the left side. Further details are presented in Table 1. Overall, 25 out of 156 (16%) patients had malignant lymph nodes according to the histopathological report. There was a 100% concordance between the side of positive lymph nodes on PSMA PET/CT and the side of positive lymph nodes on the final pathology report. However, among the 21 patients with positive lymph nodes on PSMA PET/CT, 9 (42.9%) had truly positive nodes on the final pathological report; 6 of these patients (66.7%) had undergone [68Ga]Ga-PSMA-11 PET/CT, and 3 (33.3%) had undergone [18F]F-PSMA-1007 PET/CT.

### 3.3. PSMA PET/CT Diagnostic Performance

In a visual analysis, the overall performance of radiolabeled PSMA PET/CT for identifying pathological lymph nodes showed moderate SE and PPVs, coupled with high SP and NPVs (Table 2). However, these metrics were nearly identical for [68Ga]Ga-PSMA-11 PET/CT and [18F]F-PSMA-1007, albeit slightly higher for [68Ga]Ga-PSMA-11 PET/CT.

When applying a node-to-background ratio cut-off of ≥2, the performance of [68Ga]Ga-PSMA-11 PET/CT and [18F]F-PSMA-1007 were comparable, though the latter exhibited superior SP and NPVs. Conversely, using a cut-off ratio of ≥15.5, a notable decrease in SE and PPVs was observed for [18F]F-PSMA-1007, although no formal statistical comparison was performed (Table 3).

Stratified by EAU risk group (Table 4), [18F]F-PSMA-1007 demonstrated the highest SE and SP in patients at high risk of disease when using a node-to-background ratio cut-off of ≥2. Conversely, the ≥15.5 cut-off primarily enhanced SP and PPVs.

## 4. Discussion

In this study, we analyzed two populations undergoing radiolabeled PSMA PET/CT scans, specifically with [68Ga]Ga-PSMA-11 or [18F]F-PSMA-1007, depending on the period and availability of the radiopharmaceuticals. Our results demonstrate that accuracy was slightly higher for [68Ga]Ga-PSMA-11 PET/CT than for [18F]F-PSMA-1007 PET/CT in both visual and semiquantitative analyses. However, [18F]F-PSMA-1007 PET/CT exhibited higher PPVs and NPVs, irrespective of the SUV ratio cut-off value. Notably, [18F]F-PSMA-1007 PET/CT showed the highest performance in high-risk PCa patients when analyzed per EAU risk group.

To the best of our knowledge, there are few studies comparing two different PSMA-based tracers in the staging setting, particularly focusing on lymph node metastases as the primary endpoint. Kuten et al. compared the diagnostic accuracy of [18F]F-PSMA-1007 with [68Ga]Ga-PSMA-11 in a small cohort of 16 intermediate- or high-risk PCa patients who underwent PET/CT with both tracers in a short period. Both tracers identified all dominant intraprostatic lesions, but [18F]F-PSMA-1007 detected additional low-grade prostatic lesions of limited clinical relevance [22]. Hoberük et al. reported that unspecific discrete lymph nodal uptake was more frequently observed with [18F]F-PSMA-1007 than with [68Ga]Ga-PSMA-11 PET scans (52.2% vs. 28.2%, respectively), influencing the rate of false positives [23]. A recent systematic review summarized the advantages and disadvantages of [68Ga]Ga-PSMA-11 and [18F]F-PSMA-1007 in various PCa settings. The review included head-to-head comparative series, matched-pair studies, clinical trials, and retrospective studies [24]. While the optimal use of these agents remains unclear, [18F]F-PSMA-1007 appears to have diagnostic superiority over [68Ga]Ga-PSMA-11 for identifying local recurrences, likely due to its biodistribution. However, both tracers yielded similar outcomes in staging and biochemical recurrence settings [24,25].

The physical characteristics of these radiopharmaceuticals differ significantly. [18F]F-PSMA-1007 has a longer half-life (110 min vs. 68 min) and lower positron energy, which enhances spatial resolutions and reduces blurring effects compared to [68Ga]Ga-PSMA-11. Additionally, [18F]F-PSMA-1007 can be produced centrally, facilitating its global distribution. Despite its milder SE, [18F]F-PSMA-1007 is preferable due to its feasible production and distribution.

Our study assessed the added value of semiquantitative data, specifically, the SUV ratio, for [18F]F-PSMA-1007 and [68Ga]Ga-PSMA-11 PET/CT scans in a large population of PCa patients. The identification of lymph node metastases was enhanced by using two different SUVmax ratio cut-off values (2 and 15.5). SE was mild (less than 40%) for both tracers, while SP was close to 100%. The diagnostic performance of both tracers improved with the ratio analysis compared to simple visual assessments. SP appeared to increase from 95.5% and 85.9% to 98.5% and 100%, respectively, for [68Ga]Ga-PSMA-11 and [18F]F-PSMA-1007. This study, therefore, emphasizes the impact of different SUV ratio cut-offs on the diagnostic performance of PSMA PET/CT, particularly in high-risk prostate cancer patients. Therefore, a semiquantitative analysis may reinforce visual assessments, mainly improving the SP and the NPV. This latter consideration would be helpful in avoiding an LND in patients with low-intermediate risk PCa, thus reducing subsequent side effects.

Our study has several limitations, primarily related to its relatively small sample size. Although this is one of the largest comparative studies of these two tracers to date, the limited number of true positive PET/CT results restricts this study’s statistical power, preventing firm conclusions. Additionally, the cohort included patients undergoing robot-assisted radical prostatectomy (RARP) and lymph node dissection (LND) often without radiological evidence of nodal involvement, which inherently reduced the number of pN1 cases. The limited number of true positive PET/CT results led to wider confidence intervals for SE estimates, making it difficult to draw definitive comparative conclusions between the two radiotracers. To address these limitations, we applied consistent inclusion criteria, followed standardized imaging protocols based on EANM guidelines, and used histopathology as the reference standard. Diagnostic performance measures were reported with 95% confidence intervals to transparently reflect statistical uncertainty. Furthermore, we adopted a semiquantitative approach using SUV ratio cut-offs to enhance the objectivity and reproducibility of our analysis. Another important limitation is the absence of formal statistical comparisons between [^68Ga]Ga-PSMA-11 and [^18F]F-PSMA-1007 due to the small number of true positive findings, which would render hypothesis testing underpowered and potentially misleading. As such, we focused on descriptive reporting with CI to provide a cautious but transparent interpretation of the results.

Additionally, a potential source of variability in our study is the use of two different PET/CT scanner models (Siemens Biograph 6 LSO and GE Discovery 690). Although both systems were regularly quality-checked and scans followed EANM guidelines, technical differences such as reconstruction algorithms, resolutions, and sensitivity could slightly affect SUVmax values and related ratios. This may have led to minor inconsistencies, especially when comparing tracers based on SUV thresholds. Future studies with harmonized or cross-calibrated scanners could help minimize this bias. Furthermore, a limitation of this study is the selective use of furosemide in a subset of patients undergoing 18F-PSMA PET/CT to improve the image quality in cases of intense urinary activity, which may have introduced variability despite adherence to procedural guidelines.

While we acknowledge these limitations, it is important to emphasize that our study was conducted in a real-life clinical setting, with all findings validated by a final histopathological analysis of both the prostate and lymph nodes following prostatectomy.

Finally, the single-institution nature of our study may limit the generalizability of the results. Larger multicenter studies are needed to provide more robust evidence for tracer selection.

## 5. Conclusions

Radiolabeled PSMA PET/CT is a powerful tool for the initial staging of PCa, particularly in high-risk patients, as it allows for better risk stratification and improved patient management. However, [18F]F-PSMA-1007 seems to exhibit a slightly lower SE but a relatively higher SP for nodal staging compared to [68Ga]Ga-PSMA-11, irrespective of the SUV ratio cut-off used. Correct interpretation of imaging and the use of both qualitative and quantitative PSMA PET/CT parameters can support urologists’ decision-making processes, mainly in low-intermediate categories. Sufficient powered future studies focusing on the additional utility of semiquantitative values from PSMA PET/CT are warranted.

## Figures and Tables

**Table 1:** Baseline characteristics of the overall population and by traces utilized.

		Total	[68Ga]Ga-PSMA-11	[18F]F-PSMA-1007	*p*-Value
		N = 156	N = 83	N = 73	
Age, median (IQR)		67 (60–72)	67 (60–72)	67 (61–70)	0.46 *
Family history, n (%)	No	120 (76.9)	63 (75.9)	57 (78.1)	0.92 §
	Yes	28 (17.9)	15 (18.07)	13 (17.8)	
	Missing	8 (5.13)	5 (6.02)	3 (4.11)	
Total PSA, median (IQR)		7.61 (6.00–14.3)	7.60 (6.09–15.6)	7.61 (5.38–13.0)	0.71 *
Biopsy ISUP, n (%)	1	5 (3.21)	2 (2.41)	3 (4.11)	0.28 §
	2	30 (19.2)	13 (15.7)	17 (23.3)	
	3	61 (39.1)	30 (36.14)	31 (42.5)	
	4	26 (16.7)	18 (21.7)	8 (11.0)	
	5	34 (21.8)	20 (24.1)	14 (19.2)	
Pathological ISUP, n (%)	1	1 (.641)	0 (0)	1 (1.37)	0.007 §
	2	43 (27.6)	22 (26.5)	21 (28.8)	
	3	63 (40.4)	25 (30.1)	38 (52.1)	
	4	9 (5.77)	7 (8.43)	2 (2.74)	
	5	40 (25.6)	29 (34.9)	11 (15.1)	
pT, n (%)	T2a	17 (10.9)	7 (8.43)	10 (13.7)	0.88 §
	T2b	2 (1.28)	1 (1.205)	1 (1.37)	
	T2c	60 (38.5)	32 (38.6)	28 (38.4)	
	T3a	45 (28.9)	25 (30.1)	20 (27.4)	
	T3b	32 (20.5)	18 (21.7)	14 (19.2)	
pN, n (%)	pN0	131 (84.0)	67 (80.7)	64 (87.7)	0.24 §
	pN1	25 (16.0)	16 (19.3)	9 (12.3)	
n removed nodes—right side, median (IQR)		8 (5.00–10.0)	8 (5.00–10.0)	7.5 (.005–10.0)	0.97 *
n removed nodes—left side, median (IQR)		7 (5.00–9.00)	7 (5.00–9.00)	7 (5.00–9.00)	0.97 *
n removed nodes—TOT, median (IQR)		14 (11.0–19.0)	13 (10.0–18.5)	15 (11.5–19.5)	0.39 *
EAU risk groups, n (%)	Low risk	2 (1.28)	0 (0)	2 (2.74)	0.20 §
	Intermediate risk	78 (50.0)	39 (47.0)	39 (53.4)	
	High risk	76 (48.7)	44 (53.0)	32 (43.8)	

* Wilcoxon rank sum test; § Pearson’s chi-squared test; EAU: European Association of Urology; IQR: interquartile range; ISUP: International Society of Urological Pathology; PSA: prostate-specific antigen.

**Table 2:** Overall and by-tracer performance of PSMA PET/CT using nuclear medicine report considering pathological lymph node uptake as positive or negative.

	OVERALL % (95%CI)	[68Ga]Ga-PSMA-11 % (95%CI)	[18F]F-PSMA-1007 % (95%CI)
	POS/NEG	POS/NEG	POS/NEG
SE	36.0 (18.0–57.5)	37.5 (15.2–64.6)	33.3 (7.49–70.1)
SP	90.8 (84.5–95.2)	95.5 (87.5–99.1)	85.9 (75.0–93.4)
PPV	42.9 (21.8–66.0)	66.7 (29.9–92.5)	25.0 (5.49–57.2)
NPV	88.1 (81.5–93.1)	86.5 (76.5–93.3)	90.2 (79.8–96.3)
AUC	63.4 (53.5–73.3)	66.5 (54.0–79.0)	59.6 (42.7–76.5)

AUC: area under the curve; CI: confidence interval; NPV: negative predictive value; PPV: positive predictive value; SE: sensitivity; SP: specificity.

**Table 3:** Diagnostic performance of tracers [68Ga]Ga-PSMA-11 and [18F]F-PSMA-1007 at different cut-off values of the SUV max-to-background ratio.

	[68Ga]Ga-PSMA-11 % (95%CI)		[18F]F-PSMA-1007 % (95%CI)	
	≥2	≥15.5	≥2	≥15.5
SE	37.5 (15.2–64.6)	31.3 (11.0–58.7)	33.3 (7.49–70.1)	11.1 (0.281–48.2)
SP	98.5 (92.0–100)	100 (94.6–100)	100 (94.4- 100)	100 (94.4–100)
PPV	85.7 (42.1–99.6)	100 (47.8–100)	100 (29.2–100)	100 (2.50–100)
NPV	86.8 (77.1–93.5)	85.9 (76.2–92.7)	91.4 (82.3–96.8)	88.9 (79.3–95.1)
AUC	68.0 (56.0–81.0)	66.0 (54.0–77.0)	67.0 (50.0–83.0)	56.0 (45.0–66.0)

AUC: area under the curve; CI: confidence interval; NPV: negative predictive value; PPV: positive predictive value; SE: sensitivity; SP: specificity.

**Table 4:** Comparison of the performance of the tracers stratified by EAU Risk Group.

	Low-Intermediate Risk		High-Risk		Low-Intermediate Risk		High-Risk	
	≥2	≥15.5	≥2	≥15.5	≥2	≥15.5	≥2	≥15.5
SE	40 (5.27–85.3)	20.0 (5.05–71.6)	36.4 (10.9–69.2)	36.4 (10.9–69.2)	N.V.	N.V.	50.0 (11.8–88.2)	16.7 (0.421–64.1)
SP	97.1 (84.7–99.9)	100 (89.7–100)	93.9 (79.8–99.3)	100 (89.4–100)	81.6 (65.7–92.3)	N.V.	92.3 (74.9–99.1)	100 (86.8–100)
PPV	66.7 (9.43–99.2)	100 (2.50–100)	66.7 (22.3–95.7)	68.2 (53.3–83.1)	N.V.	N.V.	60.0 (14.7–94.7)	100 (2.50–100)
NPV	91.7 (77.5–98.2)	89.5 (75.2–97.1)	81.6 (65.7–92.3)	100 (39.8–100)	91.2 (76.3–98.1)	N.V.	88.9 (70.8–97.6)	83.9 66.3–94.5)
AUC	68.5 (44.4–92.7)	60.0 (40.4–79.6)	65.2 (49.7–80.6)	82.5 (67.2–92.7)	40.8 (34.5–47.0)	N.V.	71.2 (48.6–93.7)	58.3 (42.0–74.7)

AUC: area under the curve; CI: confidence interval; NPV: negative predictive value; PPV: positive predictive value; SE: sensitivity; SP: specificity; N.V.: not valuable, due to lack of events to compare

## Data Availability

The data that support the findings of this study are available from the corresponding author upon reasonable request.

## Abbreviations

The following abbreviations are used in this manuscript:

PCa	Prostate cancer
RARP	Robot-assisted radical prostatectomy
GS	Gleason score
LND	Lymphadenectomy
LNI	Lymph node invasion
PSMA	Prostate-specific membrane antigen
PET/CT	Positron emission tomography/computed tomography
